# Fecal Microbiota Transplantation as an Alternative Method in the Treatment of Obesity

**DOI:** 10.7759/cureus.76858

**Published:** 2025-01-03

**Authors:** Shan Hemachandra, Sasanga N Rathnayake, Anne A Jayamaha, Bernadine S Francis, Dilitha Welmillage, Delvinderjit N Kaur, Hein K Zaw, Lin T Zaw, Hanna A Chandra, Maria E Abeysekera

**Affiliations:** 1 Family Medicine, Nanjing Medical University, Nanjing, CHN; 2 Gastroenterology, Nanjing Medical University, Nanjing, CHN; 3 Family Medicine, Saint Agnes Hospital, Fresno, USA

**Keywords:** bacteroidetes, clostridioides difficile, dysbiosis, fecal microbiota transplantation (fmt), gut-brain connection, gut dysbiosis, lean body mass, microbiota, obesity, ozempic for weight loss

## Abstract

Fecal microbiota transplantation (FMT) has emerged as a promising therapeutic approach for various health conditions, particularly obesity and metabolic disorders. This review examines the mechanisms underlying FMT, including its role in restoring gut microbiota diversity and enhancing immunomodulatory functions, which are essential for maintaining overall health. Recent studies indicate that FMT can significantly improve body weight and metabolic parameters, suggesting its potential as an alternative or complementary treatment to current obesity therapies. However, the effectiveness of FMT depends on several factors, including the composition of the donor microbiota, recipient characteristics, and concomitant medications or dietary interventions.

Despite its great promise, challenges such as standardized protocols, donor screening, and the need for a deeper understanding of gut microbiota dynamics remain key hurdles. Future research should focus on elucidating the specific microbial compositions necessary for optimal therapeutic outcomes and exploring personalized FMT approaches tailored to individual patient profiles. This evolving field presents exciting opportunities for innovative strategies in obesity treatment, warranting further investigation and clinical application.

## Introduction and background

Obesity is defined as BMI ≥30 kg/m². It is a complex metabolic disorder characterized by an imbalance between energy intake and expenditure, leading to excessive fat accumulation and adverse health effects. Beyond its association with lifestyle factors such as diet and physical activity, obesity is increasingly recognized as a multifaceted condition influenced by metabolic, hormonal, genetic, and environmental factors. It is strongly linked to metabolic dysfunctions such as insulin resistance, dyslipidemia, and chronic low-grade inflammation, which collectively elevate the risk of type 2 diabetes, cardiovascular disease, and non-alcoholic fatty liver disease. Moreover, obesity has been associated with an increased prevalence of certain cancers and neurodegenerative disorders, such as Alzheimer’s disease.

The global prevalence of obesity has increased rapidly over the past 30 years [1]. Out of 288 global studies involving 13,233,675 people, the overall results show the prevalence of central obesity was 41.5% (95% CI: 39.9-43.2%). The highest prevalence of obesity was observed in South America (55.1%, 95% CI: 45.8-64.3%). Males aged 15-40 years have experienced the highest rise in prevalence (25.3-41.6%), with females showing a slightly lower increase (38.6-49.7%) [2].

Recent advances have highlighted the critical role of gut microbiota in the pathophysiology of obesity and related metabolic disorders. The gut microbiota consists of trillions of microorganisms that inhabit the gastrointestinal (GI) tract and play essential roles in host physiology. These include immunomodulation, nutrient metabolism, and pathogen defense. Dysbiosis, an imbalance in gut microbiota, is implicated in several diseases, including Alzheimer’s, type 2 diabetes, inflammatory bowel disease, and obesity. Specific alterations in microbiome composition, such as an elevated Firmicutes/Bacteroidetes (F/B) ratio, have been associated with increased energy harvest from the diet, contributing to weight gain. Additionally, decreased production of short-chain fatty acids (SCFAs) and increased levels of metabolites like trimethylamine N-oxide (TMAO) have been linked to metabolic dysregulation.

The rationale for fecal microbiota transplantation (FMT) stems from the intricate methods the gut microbiota interacts with the host, including immunomodulation, metabolic processes, and pathogen defense. FMT involves introducing a diverse and healthy microbiota from a donor to the recipient’s gut to restore microbial balance. This approach has shown promise in treating recurrent Clostridioides difficile infections (CDI) and is now being explored for metabolic conditions like obesity. Understanding the complex mechanisms underlying FMT's mode of action may expand its applications and enhance patient outcomes as research on the technique advances.

The main considerations for the cause of obesity currently include people’s lifestyles and consumption behaviors. A well-recognized associative factor for the development of obesity is the health of the gut microbiome [1]. The body’s microbiome - the vast community of microbes living inside and outside the body - is essential to physiology. A “lean microbiome” has been associated with reduced body fat, whereas an “obese microbiome” is linked to increased fat storage and metabolic dysfunction [1]. Current research and marketing are geared towards the more expensive alternatives, including Ozempic (semaglutide) and Mounjaro (tirzepatide). However, the cost of these drugs is a significant burden on the taxpayers and the healthcare system as a whole. 

Research on the complex relationship between gut microbiota and metabolic health has seen a dramatic surge in recent years. The dynamic relationship between gut microbiota and obesity is explored by Lee et al. and Sehgal and Khanna [3,4], who suggest that the microbiome could be a target for intervention. FMT, with its ability to alter the gut microbiota, emerges as a promising therapeutic approach for obesity and an alternative to pharmacological interventions such as semaglutide (marketed as "Ozempic"). By restoring microbial diversity, suppressing pathogenic bacteria, and reestablishing metabolites that support host health, FMT has the potential to modulate metabolic processes and combat obesity [5]. The gut microbiome is critical in regulating the immune system, managing nutrient metabolism, and defending against infections. Dysbiosis can contribute to the etiology of metabolic disorders, including obesity.

FMT offers a novel approach to restoring microbial balance and improving metabolic health, including obesity. As a field undergoing rapid evolution, the therapeutic potential of FMT could transform obesity management, offering new hope for patients resistant to conventional therapies.

Transplanting fecal microbiota: historical background

**Figure 1:**
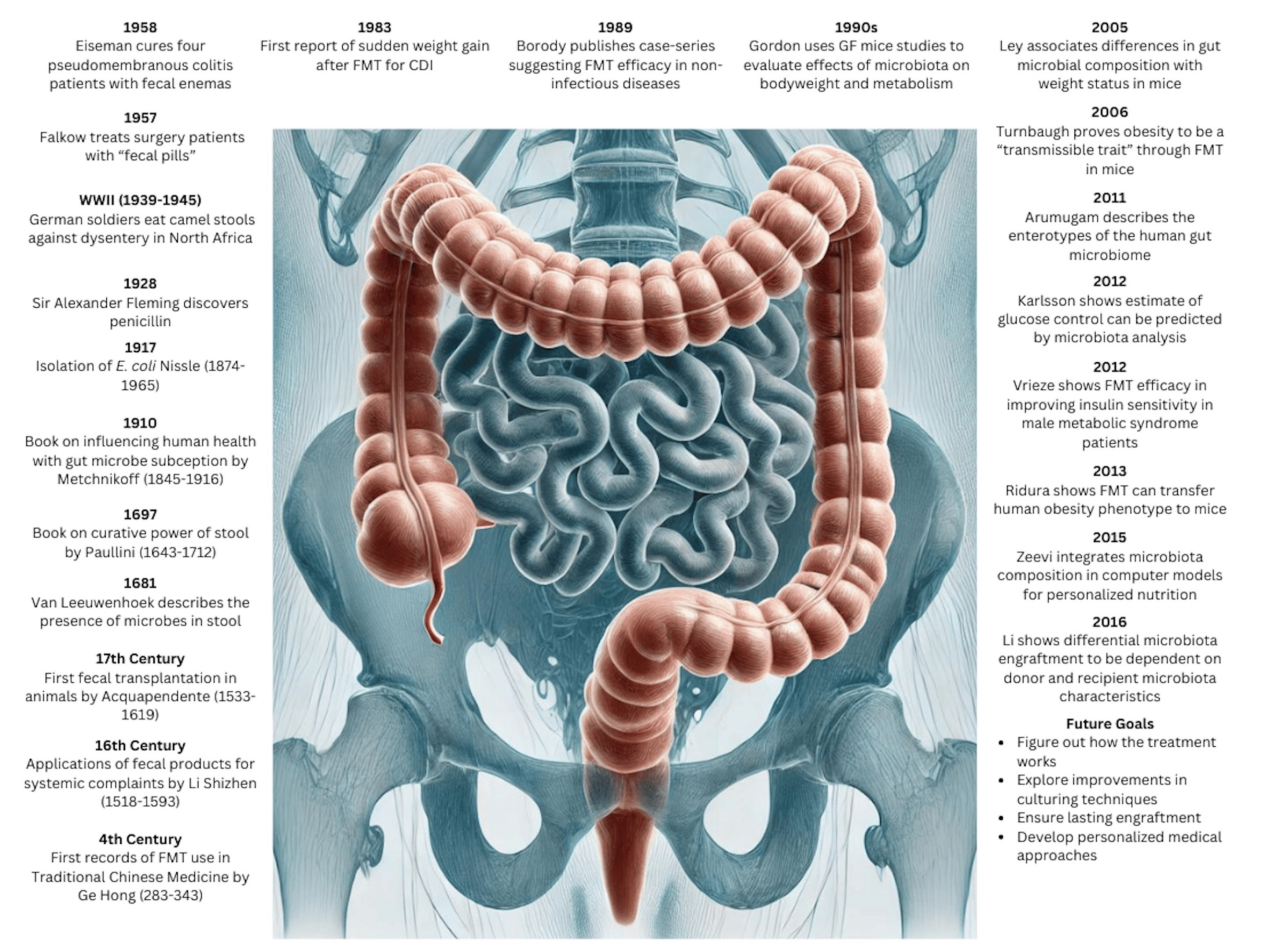
Timeline: key contributions to FMT development and research This image is reproduced with permission under an open-access license [6] FMT: fecal microbiota transplantation

The origins of FMT can be found in the oral administration of fecal material in ancient Chinese medicine, which was utilized to treat various gastrointestinal conditions. However, FMT was not well-known in Western medicine until the 20th century, when it was mostly used to treat recurring CDI. de Groot et al. [6] offer a thorough historical summary, highlighting the development of FMT as a specialized subscience. Figure 1 illustrates the historical use of FMT and how its understanding has grown over time.

FMT has attracted significant interest today as an efficacious therapeutic option for treating recurrent and refractory Clostridioides difficile (formerly known as Clostridium difficile) infections.

**Figure 2:**
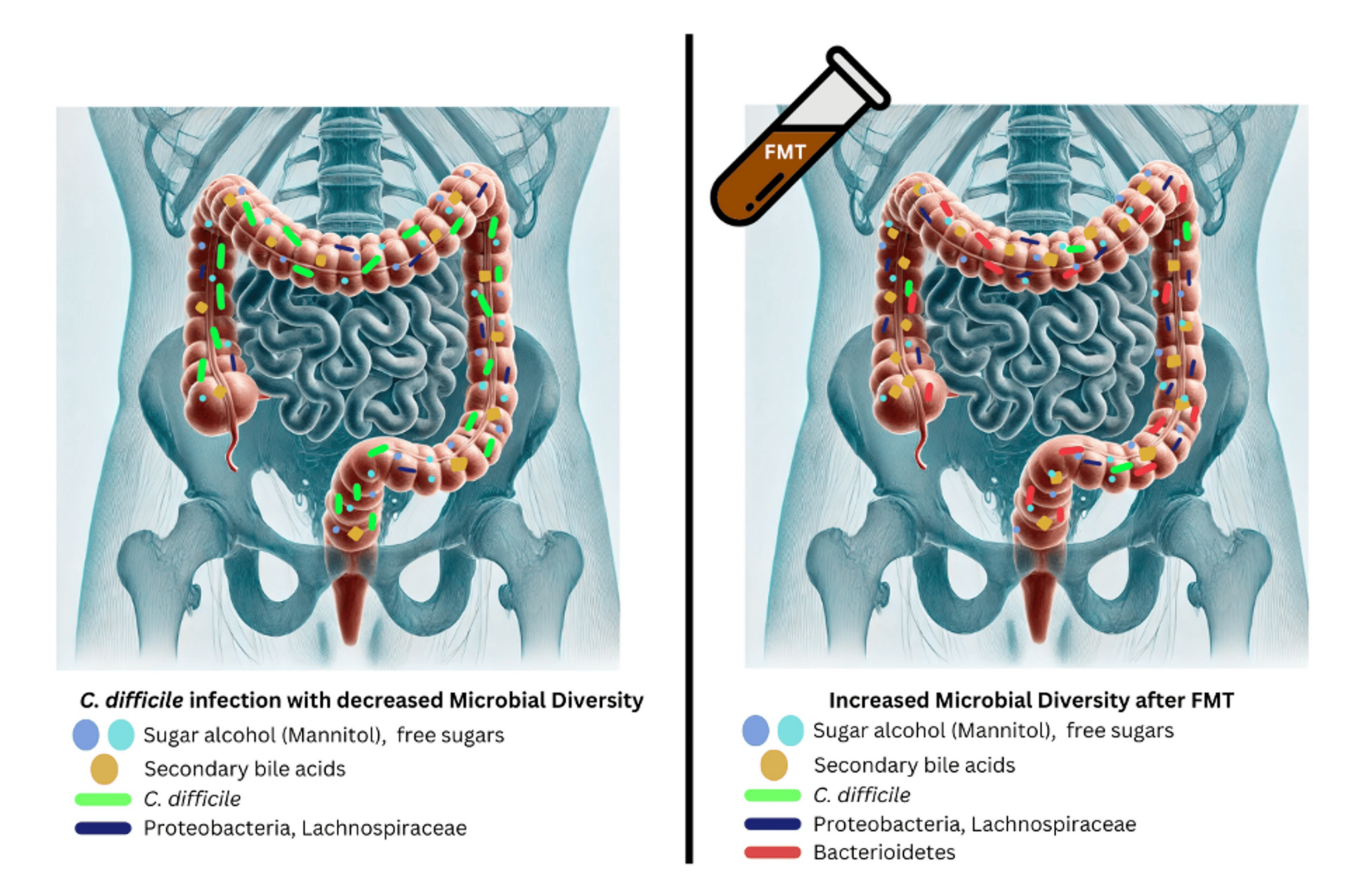
Change in gut microbiome after FMT in treatment of CDI FMT is currently used to treat recurrent and refractory CDI, and studies have shown better outcomes compared to antibiotics [7] Image credit: Author's own creation CDI: Clostridioides difficile infection; FMT: fecal microbiota transplantation

The reasons behind the effective use of FMT in the management of recurrent CDI could be attributed to changes in the microbial community, including a reduction in Proteobacteria, the restoration of microbial diversity, an increase in the production of secondary bile acids, and the exclusion of other bacteria from particular niches, which have been linked to improvements in symptoms after fecal microbiota transplantation [7]. Figure 2 illustrates the changes in microbiome composition in a patient with CDI before and after FMT.

## Review

Relationship between obesity and gut microbiome

Obesity, a global health challenge, has recently come under scrutiny due to emerging research highlighting the intricate interplay with the gut microbiota. Gut microbiota refers to over 100 trillion microorganisms, including all archaea, bacteria, microeukaryocytes, and viruses that live within a human’s GI tract.

Obesity arises due to a prolonged imbalance between dietary intake and energy expenditure, with dietary-induced modifications in the gut microbiome having a significant role in its initiation and progression. The normal gut microbiome serves specific functions in host metabolism, encompassing immunity, maintenance of the intestinal barrier, and defense against pathogens. In individuals with obesity, there are notable alterations in the composition and functions of the microbiome compared to their lean counterparts [8]. Proposed mechanisms outlining how gut microbiota may contribute to obesity development include increased energy extraction, increased plasma branched-chain amino acid (BCAA) levels, compromised intestinal barrier function, heightened endotoxemia and inflammation, changes in bile acid metabolism, and modulation of host appetite and satiety hormones [4].

Metabolic shifts associated with the modified microbiome in obesity encompass heightened energy extraction from food, increased lipogenesis, and insulin resistance. Efforts made to manage obesity through dietary interventions targeting the microbiome involve using prebiotics, probiotics, and synbiotics [8]. New studies show that colonization of the fetal GI tract occurs *in vitro* and develops throughout childhood. At age three, the microbiome composition appears similar to that of an adult. This composition changes due to a variety of factors, including race, gender, age, diet, and location of the GI tract. This difference has been observed in children, where healthier microbes are present in a child eating a healthy protein diet, compared to a child eating a Westernized diet [9].

Increased Energy Extraction

Insights from humanized mouse models have suggested that the microbiome in obese individuals exhibits enhanced efficiency in extracting energy from the diet, potentially contributing to obesity development. A systematic search of MEDLINE and Embase for case-control studies published between September 1, 2010, and July 10, 2021, revealed research comparing the intestinal microbiome in individuals with obesity and metabolic disorders to that of non-obese, metabolically healthy individuals. 

The two main bacterial phyla found in the human gut are Firmicutes and Bacteroidetes, which occupy over 90% of the gut microbiota. The Firmicutes/Bacteroidetes ratio is used as a marker to identify dysbiosis. A higher Firmicutes/Bacteroidetes ratio is associated with increased obesity and other comorbidities including inflammatory bowel disease, colorectal cancer, and type 2 diabetes mellitus (T2DM) [9]. Obesity leads to a decrease in the “good” bacteria, including *Lactobacillus, Bifidobacterium, and Akkermansia, *while levels of “bad” bacteria such as* Enterobacteriaceae, Desulfovibrionaceae, *and* Streptococcaceae *are elevated. Proteobacteria consistently emerged as the most frequently reported species associated with obesity. Bacteroidetes consistently emerged as the most frequently reported species associated with leanness [10,11,12].

Studies have identified significant associations between specific microbial genera and obesity or leanness, with variations across populations. For instance, genera such as *Faecalibacterium*, *Akkermansia*, and *Alistipes* are linked to lean phenotypes, while *Prevotella* and *Ruminococcus* demonstrate obesity associations in Western populations but lean associations in Eastern populations. Notably, *Roseburia* and *Bifidobacterium* show lean associations in Eastern populations, while *Lactobacillus* has been linked to obesity in the West. These findings underscore the complexity of microbial influences on metabolic health and emphasize the need for mechanistic studies to ascertain causal relationships. Recent meta-analyses highlight the potential for microbiota-targeted interventions to manage metabolic disorders effectively. Table 1 lists the different Phyla the relevant bacteria belong to. 

**Table 1:** Bacteria and their related phyla

Bacterial name	Phylum
Lactobacillus	Firmicutes
Bifidobacterium	Actinobacteria
Akkermansia	Verrucomicrobia
Desulfovibrionaceae	Proteobacteria
Streptococcaceae	Firmicutes
Prevotella	Bacteroidetes
Ruminococcus	Firmicutes
Roseburia	Firmicutes
Faecalibacterium	Firmicutes
Alistipes	Bacteroidetes

In another study involving FMT from discordant obese and lean adult female twin pairs in germ-free mice, notable effects were observed on total body and fat mass, along with obesity-associated metabolic phenotypes [13]. These effects were transmitted with uncultured fecal communities and corresponding fecal bacterial culture collections. Cohousing obese (Ob) and lean (Ln) mice reduced the development of increased adiposity and body mass in Ob cage mates, transforming their microbiota's metabolic profile to a lean-like state. This transformation correlated with the invasion of members of Bacteroidales from Ln into Ob microbiota, with the phenomenon being diet-dependent [13].

Compromised Intestinal Barrier Function and Inflammation

The gut microbiota is integral to maintaining the intestinal barrier. The gut microbiota composition influences the properties of the mucus layer. Key species include *Bifidobacteria*, *Lactobacillus,* and *E. coli *species which help regulate the intestinal barrier function and integrity through the maintenance and upregulation of ZO-1 and anti-inflammatory cytokines, (IL-10, IL-6, and PPAR γ), increasing the levels of occludin and E-cadherin, promoting the tight junction synthesis, and reducing bacterial translocation [13]. A compromised gut barrier leads to a compromised barrier that allows lipopolysaccharides to enter the bloodstream, which causes chronic low-grade inflammation, a key factor in the development of metabolic disturbances, including insulin resistance, which can lead to obesity [14]. The synthesis of evidence here reflects a systematic review of randomized controlled trials (RCTs) and observational studies, ensuring that only high-quality data are presented.

Bile Acid Metabolism and Obesity

Bile acid metabolism, modulated by gut microbiota, plays a critical role in energy homeostasis. Lean microbiota exhibit higher efficiency in converting primary to secondary bile acids, reducing fat storage pathways via downregulation of the Farnesoid X receptor (FXR). Dysregulated bile acid metabolism, characteristic of obese microbiota, promotes adiposity and metabolic dysfunction, highlighting the potential for microbiota-focused interventions to restore metabolic balance.

The gut microbiota of lean co-twins displayed increased abundance and expression of genes involved in transforming primary to secondary bile acids, correlating with altered bile acid levels in the host feces and serum. The study hypothesized that these bile acid alterations might affect the expression of the host FXR, a nuclear receptor regulating bile acid synthesis, transport, glucose and lipid metabolism, and inflammation. The down-regulation of FXR and its target genes in the ileum of mice colonized with the lean co-twins microbiota suggested a negative feedback loop between gut microbiota and host bile acid metabolism. Microbiota that regulate bile acids more effectively (as seen in lean individuals) can suppress pathways that contribute to fat storage. At the same time, disruption of these mechanisms can promote weight gain and obesity. The findings integrate recent systematic reviews on gut microbiota's metabolic pathways, which adhered to PRISMA guidelines by including clearly defined search strategies and eligibility criteria

Influence on Appetite and Satiety Hormones

The gut microbiota interacts with host hormones such as leptin, ghrelin, and peptide YY, directly influencing appetite regulation and energy balance. The gut microbiota from twins discordant with obesity can influence the host's metabolism and body composition in mice, with these effects being contingent on the interaction between the microbiota and the diet. Additionally, cohousing mice with different microbiota led to the invasion of specific bacterial taxa from one community to another, which was associated with the phenotypic rescue of the host [13]. The reviewed studies systematically analyzed the microbiota-diet interactions and their effects on hormonal regulation, ensuring compliance with PRISMA's checklist for comprehensive analysis.

Increased Plasma Branched-Chain Amino Acid (BCAA) Levels

BCAA catabolism is associated with metabolic disturbances leading to obesity. BCAA are essential amino acids synthesized and broken down by gut bacteria. High-quality BCAA sources include milk, dairy products, red meat, and poultry, consumed in higher quantities in the Western diet. Higher BCAA levels are related to insulin resistance, which in turn is related to obesity. A gut microbiome with a higher BCAA biosynthesis leads to increased plasma BCAA levels [15].

Gut-Brain Axis and Neurological Implications

Emerging research on the gut-brain axis reveals how gut microbiota impact brain development and function through neurohumoral communication. For example, microbial dysbiosis has been linked to altered behavioral phenotypes and neurodevelopmental disorders. Interventions targeting the gut microbiota could potentially influence neurological health, offering a promising avenue for managing metabolic and neurological disorders [16]. The systematic literature reviews included in this section adhered to PRISMA's reporting standards, ensuring a clear presentation of gut-brain axis implications supported by high-quality evidence

Other microbiota-focused treatments

Microbiota-focused treatments, such as probiotics, prebiotics, symbiotics, postbiotics, and fecal microbiota transplantation, aim to restore balance to the gut microbiota and enhance host health. While these therapies have shown promise in animal obesity models, the evidence from human studies is currently limited and inconsistent. It is crucial to explore the direct link between gut microbiota and obesity. Given the scarcity of human studies, the efficacy of microbiota-based therapies as a treatment for obesity remains uncertain, and their application should be confined to research settings [4].

Probiotics, live microorganisms offering health benefits when administered adequately, play a role in reducing obesity. Strains like *Lactobacillus* and *Bifidobacterium* impact obesity by aiding digestion, controlling hunger, reducing chronic inflammation, and regulating circadian rhythm. Thus, the general gut microbiota directly influences obesity symptoms. The literature confirms that various probiotics, whether used alone or in symbiotic mixtures, exert anti-obesity effects through species- and strain-specific mechanisms, such as gut microbiota modulation, lower insulin resistance, and enhanced satiety [17]. The various applications of live organisms and related products are defined in Table 2 below.

**Table 2:** Definitions of prebiotics, probiotics, synbiotics, and antibiotics

Term	Definition
Prebiotics	Live microorganisms that have health benefits when ingested in adequate amounts
Probiotics	Substances that are selectively used by the host microorganisms for metabolism
Synbiotics	Combination of prebiotics and postbiotics
Antibiotics	Medicine that inhibits the growth or destroys microorganisms

Changes in gut microbiome pre- and post-fecal microbiota transplantation

Bacteroidetes/Firmicutes Ratio

The Firmicutes to Bacteroidetes ratio is a pivotal marker in gut microbiota research, with implications for obesity. A systematic review included studies reporting baseline microbial compositions and outcomes after dietary or therapeutic interventions in obese versus lean participants. Inclusion criteria required detailed microbiota profiling using 16S rRNA sequencing or metagenomics, along with explicit participant dietary records. The risk of bias was assessed using the Cochrane risk-of-bias tool for randomized studies and the Newcastle-Ottawa Scale for observational studies. Before dietary therapy, obese participants manifested a reduced diversity of Bacteroidetes phylum and an elevated dominance of Firmicutes species compared to lean control counterparts. After weight loss, the relative ratio of Bacteroidetes increased whereas Firmicutes decreased, a change correlated with the weight loss percentage yet independent of variation in dietary caloric content [18,19,20,21]. Firmicutes and Bacteroidetes are the two most known microbiotas inducing this body condition. While Bacteroidetes results in inefficient calorie consumption, Firmicutes stimulates enzymes that store fat while eating calories [22]. In summary, obese people had a higher ratio in populations of firmicutes in the body. In comparison, leaner people had a higher ratio of Bacteroidetes/ firmicutes. 

Mouse Trial

Preclinical studies assessing FMT's effects on obesity were selected based on standardized protocols replicating human metabolic conditions. One such study by Park et al. (2022) implemented Jang et al.'s (2020) model. Male mice fed a high-fat diet (60% fat) for eight weeks were subjected to FMT from lean donor mice. The methodology adhered to ARRIVE guidelines, ensuring replicability:

1. Donor feces (100 mg) were homogenized in 5 mL saline and centrifuged.

2. Supernatant (100 µL) was administered orally every three days for eight weeks.

Post-transplantation, microbiome analysis revealed reduced levels of obesity-associated Firmicutes and increased beneficial bacterial species. This shift correlated with improvements in metabolic parameters, including reduced fat mass and enhanced insulin sensitivity [23,24].

Human Trial

Human trials included in the systematic review adhered to CONSORT guidelines, with randomization, blinding, and adequate sample size justification.

A 12-week double-blind study was also conducted in which metabolically uncompromised obese patients (BMI 35 kg/m^2^) were enrolled. At the same time, the FMT capsules were generated from a single lean, unrelated, healthy volunteer with a BMI of 17.5 kg/m2. The donor was a female without any known dietary restrictions. Every participant received a 72-hour premedication of 20 mg omeprazole before administering each capsule. Before the FMT, no bowel preparation or antibiotics were given. 

Overall, it was discovered that FMT using oral capsules from a lean donor was safe and well tolerated and, at week 12, produced a bile acid profile and microbiota similar to those of a lean person. One crucial point is the development of the bile-hydrolyzing genus Faecalibacterium, which is not found in the obese microbiome profile and may have decreased primary bile acids. Bile acids control glucose, lipid, and energy balance and aid in fat absorption. However, there was no significant change in the BMI (mean) at week 12 [24].

A study was conducted in southern China to investigate the effect of washed microbiota transplantation (WMT) in metabolic syndrome (MS) patients. WMT is a process that further purifies the FMT to reduce harmful effects. Clinical data of patients treated with one to three treatments of WMT were collected out of a total of 237 patients, which included 195 non-MS patients and 42 MS patients. The results are shown in the table below, indicating that WMT can be put forward as a new clinical approach for the treatment of obesity and MS by regulation of gut microbiota [25]. The short-term and medium-term changes in MS following WMT are outlined in Table 3 below.

A study investigating the effects of washed fecal microbiota transplantation (WMT) on obesity patients was conducted with 166 patients including 114 normal-weight patients and 52 overweight (OW) patients.

**Table 3:** Short- and medium-term changes in metabolic syndrome after WMT This table is reproduced with permission under an open-access license [26] WMT: washed fecal microbiota transplantation

Parameter	Short-term results	P-value (short term)	Medium-term results	P-value (medium term)
Fasting blood glucose (FBG)	Decreased	0.023	Decreased	0.048
Triglycerides (TG)	Decreased	0.030	-	-
Systolic blood pressure (SBP)	Decreased	0.026	-	-
Body mass index (BMI)	Decreased	0.031	Decreased	0.048
High-density lipoprotein (HDL-C)	Increased	0.036	-	-
Total cholesterol (TC)	-	-	Decreased	0.022
Low-density lipoprotein (LDL-C)	-	-	Decreased	0.043
Non-HDL-cholesterol (non-HDL-C)	-	-	Decreased	0.024

The results showed that WMT notably improved the BMI, blood glucose, blood lipids, and other indicators of OW patients in the short duration of one month and medium duration of two months. WMT also altered OW patients' gut microbiota composition and diversity, enhancing the abundance of beneficial bacteria. Moreover, WMT modulated the sphingolipid metabolism of OW patients, which may play a role in regulating energy homeostasis and inflammation. Therefore, the study suggested that WMT is a safe and effective therapy for obesity and its complications [27].

A systematic review article on the effects of FMT on obesity and MS including three randomized placebo-controlled studies with 76 patients reported that FMT improved peripheral insulin sensitivity in six weeks for patients receiving FMT compared to the placebo control. HbA1c levels also showed lower levels in FMT patients in six weeks. It also stated that FMT altered the patients' gut microbiota composition and diversity, increasing the abundance of beneficial bacteria [27]. A meta-analysis of RCTs evaluating FMT's impact on metabolic parameters was conducted, including 154 patients from six studies. Short-term benefits (two to six weeks) included improved HbA1c and HDL cholesterol levels. Another review highlighted improved peripheral insulin sensitivity and reduced inflammation in FMT recipients compared to placebo controls [28].

Mechanism of fecal microbiota transplantation

Steps of Fecal Sample Preparation

1. Test sample for contaminants and pathogenic species

2. Stool samples are mixed with a blender or centrifuge to homogenize samples in a correct ratio. The stool, saline, and glycerol ratio is 25% for stool, 65% for saline, and 10% for glycerol. 

3. Filter and resuspend the mixture till it is fully homogenized. 

4. Store in cryotolerant containers at -80°C till needed. 

5. When needed for use, load into capsules or deliver through endoscopy, colonoscopy, enteric nasal tube, or enema. 

**Figure 3:**
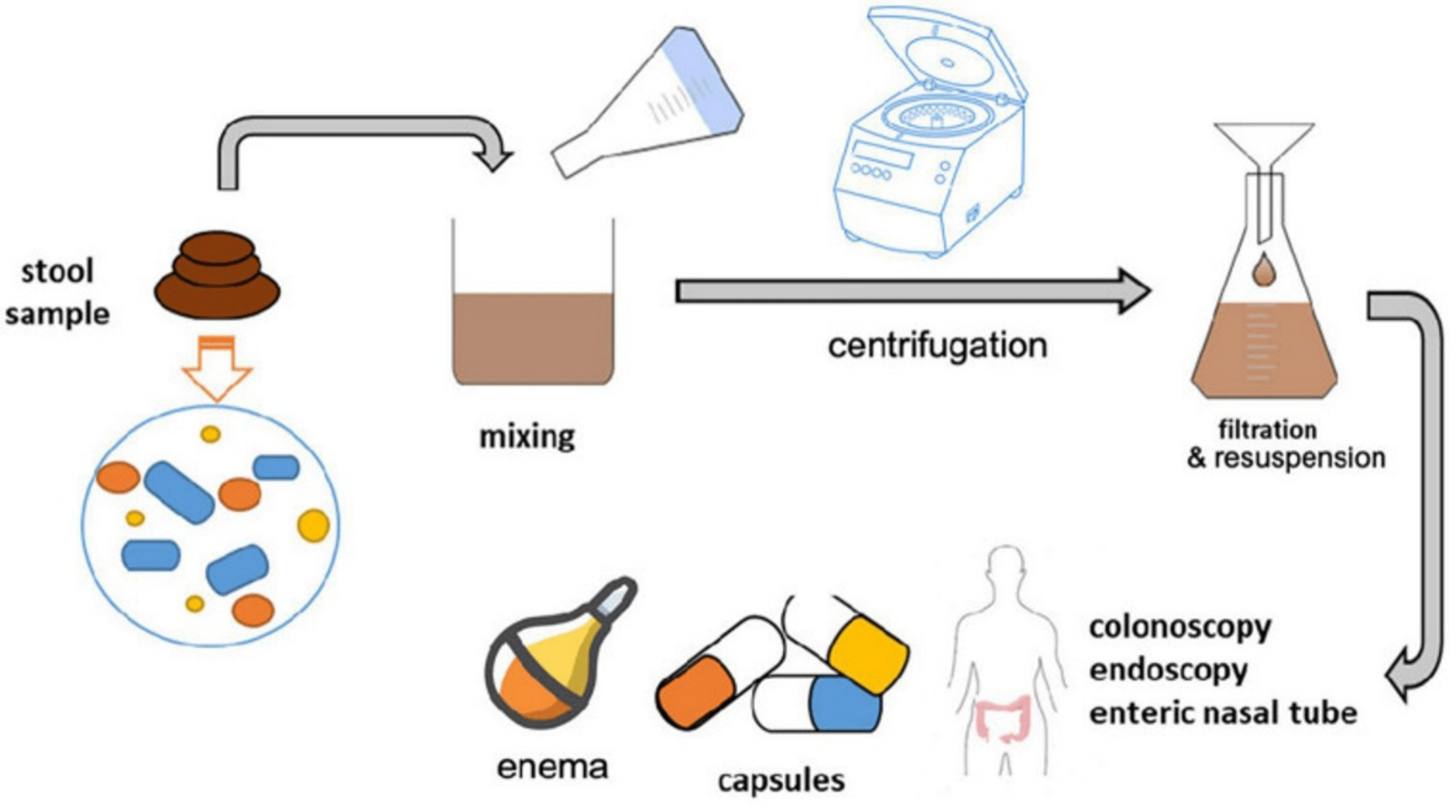
Steps of fecal sample preparation This image is reproduced with permission under an open-access license [9]

This process is visualized in Figure 3 below. 

Restoring a varied and healthy gut microbiota in the recipient is the mechanism behind FMT. Numerous studies have been conducted on the vital function of gut microbiota in preserving health and avoiding disease. Gaining knowledge about the methods via which FMT works might help better understand how it might be used in different situations. An update on FMT in 2015 was given by Kelly et al. (2015) [7], who described the indications, methods, mechanisms, and forecast for the future. This thorough analysis investigated the possible pathways by which FMT affects the gut flora and associated host reactions.

Furthermore, the gut microbiota's immunomodulatory capabilities are vital to the FMT mechanism. The changes in gut microbiota composition may affect immune function as they influence the host immune system. FMT may work by modifying the recipient's immune responses, resolving inflammation, and returning the immune system to homeostasis. Ongoing research into the specific immunomodulatory effects of FMT could inform the development of targeted therapeutic approaches.

Fecal microbiota transplantation as a substitute for traditional obesity treatment

Conventional obesity treatments-pharmaceuticals, surgical interventions, and lifestyle modifications-are effective but often accompanied by side effects or limited long-term efficacy. Recent meta-analyses of semaglutide use demonstrated substantial weight and BMI reductions, but adverse effects like nausea and diarrhea were common [29,30]. 

Eight new meta-analysis studies involving 4567 patients on the use of Ozempic (semaglutide) on obese patients revealed significant body weight loss [mean difference (MD): −10.09%; 95% CI: −11.84 to−8.33; p˂0.00001] with an even larger reduction in BMI (MD:−3.71 kg/m^2^; 95% CI: −4.33 to−3.09; p˂0.00001) and waist circumference (MD: −8.28 cm; 95% CI: −9.51 to−7.04; p˂0.00001). There was also a notable reduction in patients’ blood pressure, lipid levels, and inflammatory markers. Adverse effects of the use of semaglutide include nausea and diarrhea [31].

The studies conducted by Aron-Wisnewsky (2019) provide robust data that suggests FMT may be a viable treatment option for obesity and diabetes in the future [31,32]. It provides information about the revolutionary potential of FMT, which is seen as a paradigm shift in obesity therapy. Significant research on the effects of FMT with oral capsules in obese patients is provided by Allegretti, with implications for practical implementation in real-world settings.

In summary, FMT's evolutionary path from traditional medical applications to modern ones has established it as a cutting edge in the fight against obesity. Using FMT instead of traditional therapies creates new opportunities for individualized and focused treatment approaches. FMT is leading the way in uncovering the complex relationships between gut microbiota and obesity, providing hope for a paradigm shift in treating obesity.

Factors affecting the success of fecal microbiota transplantation

Several factors contribute to potentially impacting and modulating the success of FMT.

Microbiota Engraftment Upon Donor FMT

Studies have shown that the FMT receiver's microbiota composition modifies towards that of the healthy donor within one week after intervention [33].

It is observed that FMT may act in normalizing microbiota dysbiosis, which is suggested by an increase [34] and even a restoration in microbial gene richness (MGR) post-FMT. However, this observation has only sometimes been reproduced [35].

In addition, the Single Nucleotide Variants (SNV) analyses showed that the dominant bacterial strains in the donor and the receiver were not always similar, even though they did have a resemblance one month post-FMT [36], which was further confirmed three months post-FMT [37]. Therefore, it is suggested that gut microbiota composition continues to change post-FMT with time. 

To potentially induce the confounding effects of the donor's microbiota engraftment into the receiver, some studies have used antibiotics before the FMT procedure. A recent study without antibiotic use pre-FMT showed significant variability in FMT engraftment among individuals [38].

FMT induced a marked modification of the receiver’s microbiota compared to placebo, which initially lasted for at least three months. After that, the receiver's composition started to lose similarity with the donor’s composition. In addition, in all receivers, post-FMT donor-specific species were only moderately elevated yet higher than those observed post-placebo FMT and were specific to donor pairs [38]. Therefore, a durable coexistence of strains of bacteria present in the receivers and those transferred from the donor was induced by FMT. A significant strain replacement exists in the receiver, yet with variability from one receiver to the other [38], which could partially explain the variable response of FMT [35,39].

Overall, it is suggested by these results that the colonization of a new microbiota, which FMT induces, can interact with that of the receiver and is different from that of the donor. Hence, it emphasizes the importance of improving the engraftment of FMT according to the microbiota receiver and maximizing clinical outcomes via dietary or antibiotic treatment before donor FMT, which increases donor bacterial engraftment. 

Composition of Donor’s Microbiota and Donor FMT Success

The notion of super-donors that has emerged from several studies (e.g., in the context of IBD) is essential in evaluating the donor's microbiota composition in the success of FMT. In the context of ulcerative colitis, seven in nine patients who entered remission received their FMT from one donor [40].

Evidence from an FMT study in IBD patients that used pooled feces from multiple donors showed that those who entered remission had received FMT from a single donor who is suggested to be a super-donor [41]. However, all patients have been on TNFα blockers before receiving FMT, which might have affected the outcome of FMT. Therefore, further investigations are required to confirm whether the notion of a super donor is valid in the metabolic context. 

Even though there is evidence to suggest that high diversity in donor's microbiota has been associated with good response in IBD remission [42], it is still not confirmed whether a donor is considered a suitable donor for every disease type mainly because the microbiota of the donor has to be enriched in specific strains that are reduced or even absent in a specific disease to induce a good response in that specific disease. Hence, it warrants further investigations.

Effects of Concomitant Drug Use and Donor FMT Effect

Examining the intake of glucose-lowering drugs by FMT receiver is critical when using FMT to treat Type-2 Diabetes (T2D). Change in the composition of gut microbiota, together with an increase in beneficial bacterial strains and pro-inflammatory strains like E. coli, is induced by the first line of anti-diabetic treatment, Metformin [43,44]. It was observed that there was a shift in microbiota composition in T2D patients with or without metformin, along with elevated E. coli and a reduction in butyrate-producing bacteria, while not impacting MGR [45]. Another study done using germ-free mice identified a microbiota signature strongly related to metformin, which reproduced some of the beneficial effects of the molecule post-FMT experiments [46]. It is suggested that evaluating the effect of FMT in T2D patients who receive FMT from healthy donors is difficult since patients with T2D who are on one or more glucose-lowering drugs may have a different impact on gut microbiota composition as this has a possibility of interfering with the engraftment of the donor’s gut microbiota post- donor FMT.

The concomitant drug use for co-existing illnesses such as T2D in an obese patient may be worth looking at in the future; however, currently, there is a lack of evidence in this area.

Effects of Concomitant Dietary Intervention and Donor FMT Effect

Short and long-term food intake habits can impact gut microbiota profile [47]. Moreover, it has been found that there are at least partial changes in the composition of gut microbiota induced by acute modification of dietary intake [47,48].

FMT experiments using rodents have demonstrated that dietary intake from FMT receivers might impact the clinical effects of FMT, as discussed in [49]. A differential metabolic and corpulence improvement was induced from the diet administered to the germ-free receiver mice when given FMT from obese or lean twins [50]. However, a standard recommendation regarding the optimal diet that would maximize the beneficial therapeutic effect and engraftment of bacterial strain does not exist. In addition, the donor's diet might also have an impact on the success of FMT, as suggested by a study done recently using FMT from vegan lean donors in which a switch in the receiver's gut microbiota composition towards that of the vegan donor was observed (with some individual variability) [39]. It was essential to observe that no functional modification accompanied the switch. The potential cause for this could be the interindividual variability of receivers' microbiota composition and the diet consumed by the receiver, which remained the same pre and post-FMT [39].

Allergic Reactions or Immune Responses to Transplanted Fecal Matter

Allergic reactions or immune responses to the transplanted fecal matter are potential shortcomings of FMT. Introducing foreign microorganisms via MT can activate the recipient's immune system, leading to an immune response or allergic reaction. These reactions range from mild symptoms like fever, rash, or gastrointestinal discomfort to more severe outcomes like anaphylaxis [51].

Considering the varying immune responses of individuals, it is essential to thoroughly assess potential risks before opting for fecal matter transplantation [7].

Challenges and future considerations related to fecal microbiota transplantation

The safety and effectiveness of FMT are clinically evident; however offering more solutions to mitigate the recognized issues hindering the success of FMT, such as the risk of disease transmission from the donor to the recipient, acceptance of patient, undesirable outcomes, and uncertain impacts on the immune system of the recipient, are required. 

To standardize donor screening and set up a clear set of protocols for monitoring adverse events, an FMT registry must be in place to collect long-term data and follow-up outcomes and complications. Whether diet modification post-FMT could improve FMT response has to be investigated in the future [32].

Studies conducted over the last few years have provided sufficient knowledge regarding the bacterial population in the human intestine; however, there needs to be more evidence available about the function of intestinal bacteria and the viral and fungal composition in the gut. Moreover, the highly dynamic composition of live microbiota that is sensitive to external factors such as diet and drugs is another uncertainty attached to the success and outcomes of FMT [47].

Therefore, when designing research studies in the future, it is suggested that researchers focus on identifying and defining gut microbiota and their function and more precise manipulation of gut microbiota. Personalized FMT tailored for different patients and conditions based on variability and disease genotypes/phenotypes of the host should be anticipated in the years to come [13].

Fecal microbiota transplantation shortcomings and other gaps

Fecal microbiota transplantation (FMT) has emerged as a promising treatment for recurrent Clostridioides difficile infection (CDI). However, despite its effectiveness, FMT has several shortcomings and gaps that must be addressed before widespread use for the treatment of obesity. Firstly, the lack of standardized protocols and guidelines poses a challenge in ensuring consistency and safety of the transplantation process [52].

Lack of standardization in sample collection, DNA extraction, and data analysis hampers the comparability of research findings across studies [53]. The limited availability of appropriate donors and the potential risk of transmitting unknown pathogens remains a concern [4,54]. Furthermore, the long-term effects and potential complications of FMT are still poorly understood, warranting further research in this area. 

Variation in donor selection and screening processes is another significant drawback of FMT. Currently, FMT procedures have no standardized donor selection and screening protocol. Some institutions rely on self-reported questionnaires to assess potential donors for risk factors. Standardization raises concerns about the potential transmission of infectious diseases or unknown pathogens during FMT procedures.

Incomplete knowledge of the specific microbial composition required for optimal results poses a significant challenge in FMT. The intricate and dynamic nature of the gut microbiota makes it challenging to pinpoint the exact bacterial strains necessary for therapeutic success. Allegretti et al. (2020) [32,55,56] noted that current research needs a comprehensive understanding of the specific microbial components that contribute to various disease states and their respective responses to microbial restoration therapies.

Consequently, this knowledge gap hinders the ability to tailor transplantation procedures to individual patient needs. Challenges in identifying and characterizing diverse microbial communities are primarily attributed to the limitations of current laboratory techniques and the need for comprehensive databases for comparative analysis. The complex nature of microorganisms, with their high genetic diversity and dynamic interactions, requires advanced sequencing technologies and bioinformatics tools for accurate identification and classification [46,57,58].

## Conclusions

FMT has emerged as an innovative treatment option, especially for recurrent CDIs, with potential applications in obesity and metabolic disorders. By restoring a diverse and healthy gut microbiota, FMT can significantly alter gut composition, influencing immune responses and reducing inflammation, thereby enhancing overall health. However, challenges persist, including the need to standardize FMT protocols and address donor safety, as variations in microbiota can affect treatment outcomes. Personalization of treatment, taking into account factors such as previous antibiotic use and dietary habits, is crucial for optimizing FMT efficacy. Looking ahead, ongoing research is essential to refine FMT methodologies and investigate its broader therapeutic potential while addressing associated risks, such as immune reactions to the transplanted material. Establishing clear guidelines for donor selection and patient monitoring will be vital for the successful integration of FMT into clinical practice. Although FMT shows great promise in addressing gut-related health issues, further studies are needed to optimize its benefits and ensure safety in diverse patient populations.
